# Inspired and Effective: The Role of the Ideal Self in Employee Engagement, Well-Being, and Positive Organizational Behaviors

**DOI:** 10.3389/fpsyg.2021.662386

**Published:** 2021-05-20

**Authors:** Hector A. Martinez, Kylie Rochford, Richard E. Boyatzis, Sofia Rodriguez-Chaves

**Affiliations:** ^1^Organizational Behavior Department, INCAE Business School, Alajuela, Costa Rica; ^2^Management Department, The University of Utah, Salt Lake City, UT, United States; ^3^Organizational Behavior, Case Western Reserve University, Cleveland, OH, United States; ^4^Research Department, INCAE Business School, Alajuela, Costa Rica

**Keywords:** ideal self, meaningfulness of work, psychological well-being, employee engagement, OCB helping, OCB voice, role theory, self-regulation theory

## Abstract

This study explores the efficacy of a specific tool – the articulation of the ideal self – in job engagement, psychological well-being, and organizational citizenship behavior. We hypothesized that employees who can visualize their jobs as part of their ideal self – in particular how it helps in its development and realization – would feel higher levels of engagement and fulfillment in their lives, as well as engage in greater amounts of helping and voice OCB. A total of 239 full time employees from five companies in Costa Rica filled out the ideal self questionnaire, the job engagement, and psychological well-being surveys, and were evaluated by their peers on task behavior and helping and voice OCB. Results of the SEM model showed that the ideal self was positively related to job engagement, psychological well-being and helping and voice. These findings contribute to the research on the impact and importance for organizations to help employees find meaningfulness in their work.

## Introduction

Scholars have noted that the employer-employee relationship has evolved from a long-term relationship, characterized by “employees offering loyalty, trust and commitment in exchange for job security, training and development, promotion and support from their employers” (Cartwright and Holmes, 2006, p. 200), to a more transactional employment contract (Herriot and Pemberton, 1995; Rousseau, 1995; Cartwright and Holmes, 2006), in which – in exchange for higher pay – “employees are expected to work longer hours, take on greater responsibility, be more flexible and tolerate continual change and ambiguity” (Cartwright and Holmes, 2006, p. 200). This shift in the work relationship has led some scholars to conclude that employee cynicism is the new paradigm that defines the work relationship (Cartwright and Holmes, 2006).

In this more transactional work relationship, several employment issues have fallen by the wayside, and statistics seem to provide evidence that one of these is the meaningfulness that employees find in their work. The most recent Gallup poll suggest that only 34% of US employees describe themselves as feeling engaged at work, while the rest report that they are either unengaged (53%) – or worse – actively disengaged (13%) (Harter, 2018). Yet the meaningfulness of work is important for organization and employees because – while it is a subjective determination of the significance in their job role (Steger et al., 2012) – employees who find meaning in their work have higher levels of job satisfaction (Wrzesniewski et al., 1997), well-being (Ryff, 1989; Ryan and Deci, 2001; Rosso et al., 2010; Allan et al., 2014; Hooker et al., 2019), individual performance (Hackman and Oldham, 1980; Wrzesniewski, 2003), in-role behavior (Wrzesniewski and Dutton, 2001; Bunderson and Thompson, 2009; Berg et al., 2010), extra-role behavior (Bateman and Organ, 1983), and have higher quality relationships at work (Chalofsky, 2010; Rosso et al., 2010; Fairle, 2011; Shuck and Rose, 2013). However, its emergence is “nuanced and individually offered” (Shuck and Rose, 2013, p. 3), and “cannot be demanded, artificially created, or inflated” by the organization (Shuck and Rose, 2013, p. 3). Additional to this, the sense of meaning at work has been theorized to be dependent on the individual's subjective evaluation (Schnell et al., 2013). As such, contemporary organizations sometime view the meaning employees find in their work as being outside of their purview, yet they still expect “more engagement than they […] are willing to invest in developing” (Shuck and Rose, 2013, p. 3).

Following Boyatzis and Akrivou (2006) proposition that individuals who can better articulate how their jobs are a part of their ideal self – as well as defining the role it plays in its development and realization – would have higher levels of engagement and fulfillment in their lives, in this paper, we explore the efficacy of a specific tool – the articulation of the ideal self – to predict a variety of positive employee outcomes including job engagement, psychological well-being, and organizational citizenship behavior (OCB). It has been proposed that the ideal self may be particularly relevant to this topic because “how individuals see themselves and how they are oriented toward the activity of work plays a crucial role in the meaning of that work” (Rosso et al., 2010, p. 99). Additionally, while the work relationship may have become more transactional, employees are also more entrepreneurial in their development, focusing more on self-managing their protean careers (Arthur and Rousseau, 1996; Cartwright and Holmes, 2006), or crafting their jobs to fit their needs, desires and strengths (Wrzesniewski and Dutton, 2001). Given this, perhaps helping employees visualize how their jobs help them to develop into and maintain their most desired version of themselves may hold some part of the answer to the engagement crisis.

Relying on self-regulation theory (Harlow and Cantor, 1996) and role theory (Katz and Kahn, 1966), we argue that employees who have a more clear, meaningful, and inclusive ideal self are more able to visualize the role of their job in realizing that vision (Boyatzis and Akrivou, 2006). This in turn, will lead to higher levels of job engagement and well-being, and—as part of the way they bring their ideal future into reality—expand their role breadth (McAllister et al., 2007) at work to engage in higher levels of in-role and extra-role behaviors. This argument is tested using a sample of 239 full-time employees from five firms in Costa Rica. Data was collected over a four-month period with both self and other ratings.

Our findings indicate that the ideal self shows a significant and positive relationship with job engagement, psychological well-being, and two different types of citizenship behavior: helping and voice. Our findings suggest that if organizations can encourage and help employees to better visualize the role of their work in realizing and maintaining their ideal self, both organizations and employees will reap the rewards of meaningful work.

## Literature Review

### The Self-System and Self-Regulation

The self-system is a collection of possible self-representations that are impacted by time (i.e., past, present and future selves), domain (i.e., actual, ideal and ought) and standpoint (i.e., own vs. others) (Higgins et al., 1986; Markus and Wurf, 1987). These possible selves are described as self-schemas that derive from representations of past selves and include representations of future selves (Markus and Nurius, 1986), capturing specific, personally poignant hopes, fears, and fantasies (Markus and Nurius, 1986). While they include past images of the self, as well as lived experiences, they are dynamic through the inclusion of a sense of what is possible for the self (Markus and Nurius, 1986). Some possible selves capture the form of our desires for mastery, power, or affiliation, while others are bleak, sad, or tragic reminders of what needs to be avoided (Markus and Nurius, 1986). Some possible selves work directly to energize or activate behavior, while others do not appear to instigate behavior directly, but are mediated by what is believed to be possible and important (Markus and Nurius, 1986). As such, possible selves are important for two main reasons: they provide context to interpret and evaluate current and past selves, and also motivate behavior (Hoyle and Sherrill, 2006).

### Self-Regulatory Possible Selves

The possible selves that are seen as most likely to impact behavior are termed self-regulatory possible selves (Oyserman et al., 2004). Self-regulation is a “fundamental activity of the self-system, the organized, dynamic, and causal constellation of thoughts, feelings, and motives that constitutes people's experience of themselves” (Hoyle and Sherrill, 2006, p. 1673). Self-regulatory possible selves provide a self-defining goal and include specific behavioral strategies for pursuing the goal (Hoyle and Sherrill, 2006), and through a feedback process of action, comparison and behavior adjustment, individuals “more closely approximate the reference value” (Carver and Scheier, 1990, p. 19). These self-regulatory possible selves work as guides that individuals pursue or avoid (Hoyle and Sherrill, 2006). Those to be pursued are desirable, positive and can be based on observations of other people rather than personal experience (i.e., hoped-for selves) (Hoyle and Sherrill, 2006). For example, a hoped-for possible self provides an image that captures motivation (i.e., a general drive or inclination to do something) for self-regulation (i.e., the self's capacity for altering its behavior) toward the future self (Baumeister and Vohs, 2007).

### The Ideal Self and Self-Regulation

One of these possible selves – the ideal self – has been identified as playing a prime role in self-regulation (Carver and Scheier, 1990). The ideal self is a specific type of possible self that can be described as a “future best self,” representing a version of ourselves that is consistent with not only what is most important to us (i.e., values, goals and experiences), but also what we find as aspirational and inspirational, representing the future self that one most desires to become (Markus and Nurius, 1986). It is “an evolving, motivational core within the self, focusing a person's desires and hopes, aspirations and dreams, purpose and calling” (Boyatzis and Akrivou, 2006, p. 625). It is “partially conscious and partially unconscious, varying from individual to individual” (Boyatzis and Akrivou, 2006, p. 625), and finding congruences between the “real” or actual self and the ideal self leads to “attaining something desired” or “acquiring rewards” (Carver and Scheier, 1990, p. 32), leading Boyatzis and Akrivou (2006) to propose that a healthy and robust ideal self can energize managers and lead them to feel a general satisfaction in their lives. They explain that the ideal self leverages the motivational power of a salient and meaningful vision of what could be, playing an “executive or motivational function” monitoring and guiding actions and decisions to ensure self-satisfaction, leading to behaviors that move the current self toward the realization and development of the ideal self (Boyatzis and Akrivou, 2006, p. 625). The vision of the ideal self helps to organize and direct the will to change, embedding it “with positive affect from within the person” (Boyatzis and Akrivou, 2006, p. 625). It is because of this dynamic that positive psychology has identified the ideal self as a prime mechanism for motivation, self-fulfillment and general satisfaction in one's life (Boyatzis and Akrivou, 2006), as active dreaming, thinking, imagining, and investing in the realization of this inspirational ideal self activate positive emotions and vitality in individuals.

### The Ideal Self and How Employees Feel

#### Job Engagement

Kahn defined job engagement as “the simultaneous employment and expression of a person's “preferred self” in task behaviors that promote connections to work and to others, personal presence (physical, cognitive, and emotional) and active, full performances” (Kahn, 1990, p. 700). When experiencing engagement, employees “harness their full selves in active, complete work role performances by driving personal energy into physical, cognitive, and emotional labors” (Rich et al., 2010, p. 617). Engaged employees are described as focused in their role performances, as well as being present psychologically, attentive, and feeling connected (Rich et al., 2010). They are open to themselves and others, connected to work and others, and bring their complete selves to perform (Kahn, 1992).

Kahn (1990) proposed that there were conditions that fostered the willingness for an employee to engage in their work roles. These included an individual's perception of their work contexts, as well as their personal characteristics. Kahn identified three psychological conditions: (1) salience of the work role; (2) safety of the environment; (3) and resource availability (Rich et al., 2010). Prior research has linked the ideal self to the similar construct of career commitment (Buse and Bilimoria, 2014). Research on the power of positive imagining “indicates that we can access and engage deep emotional commitment and psychic energy if we engage our passions and conceptually catch our dreams” (Boyatzis, 2006, p. 614). As such, an expressed ideal self that connects the developmental impact of one's current job in the vision of the future ideal self would impact employee engagement by providing clear salience of the work role, provides evidence of psychosocial and psychological safety (Edmonson, 1999; Dollard and Bakker, 2010) in the environment, and through positive affect, provides more psychological resources (Fredrickson, 2001; Boyatzis, 2006).

Thus, if employees are able to visualize their work roles within their ideal self, either as a path toward or a component of their most desired self-regulatory possible self, then employees will feel engaged in their work not only because it will lead to better job performance, but also because it will be an investment toward the realization and development – or maintenance – of their ideal self. It is expected that an individual who feels that their ideal self is holistic and inspirational – inclusive of their job – will have greater levels of job engagement.

*Hypothesis 1: The ideal self is positively related to job engagement*.

#### Psychological Well-Being

Well-being refers to “optimal psychological functioning and experience” (Ryan and Deci, 2001, p. 142), and fits within the debate about the definition of “what constitutes “the good-life”” (Ryan and Deci, 2001, p. 142). Definitions of well-being fall into two ancient philosophical views: The first, labeled hedonism, equates well-being with pleasures, while the second, termed eudaimonism, determines that well-being moves beyond happiness and consists of realizing one's “true nature” or potential (Ryan and Deci, 2001). Researchers have found that while measures of hedonic – or subjective well-being (SWB) – and eudaimonic well-being – psychological well-being (PWB) – are strongly correlated, they capture distinct types of experience (Waterman, 1993). “PWB tradition draws heavily on formulations of human development and existential challenges of life” (Keyes et al., 2002, p. 1008).

Ryff (1989) has proposed that PWB is made up of six distinct dimensions: self-acceptance, positive relations with others, environmental mastery, autonomy, purpose in life and personal growth. PWB research has looked at well-being as outcomes in response to personal projects (McGregor and Little, 1998) and work aspirations and achievements (Carr, 1997). However, the meaningfulness of these goals and aspirations impacts the level of well-being. For instance, Sheldon and Elliot (1999) found that goals aligned with intrinsic and autonomous motivations were associated with greater well-being. This effect was significantly weaker when the attained goals were not autonomous. Nix et al. (1999) found that whereas successful goal pursuits led to happiness, it was only when the pursuits were autonomous that success yielded vitality. Furthermore, McGregor and Little (1998) found that, whereas perceived efficacy was linked to happiness, the relative integrity of goals was linked to meaningfulness.

It would be expected that the ideal self plays a role in PWB for several reasons. The conscious search to express the ideal self aligns and incorporates several of the six dimensions of PWB as expressed by Ryff. For example, Boyatzis' intentional change theory notes that the ideal self is grounded on identifying and choosing one's desired developmental path (i.e., purpose in life and autonomy), which requires rejecting the “ought self” (Higgins, 1987) (i.e., self-acceptance), to generate the necessary energy for change toward the ideal self (i.e., personal growth. As such, individuals who have worked to discover and express their ideal self would experience greater levels of well-being because people's experiences of well-being are “shaped by attributes of their personal goals and their motives for pursuing them” (Ryan and Deci, 2001, p. 143). From this perspective, PWB is a result, in large part, from the satisfaction of needs for autonomy, competence, and relatedness (Reis et al., 2000). Because the ideal self is both a vision of one's most integral desires and a path for its development, then it is expected that the ideal self will have an impact on employee PWB.

*Hypothesis 2: The ideal self is positively related to PWB*.

### The Ideal Self and Employee Behavior

#### Job Performance and Role Behavior

Job performance research is founded on (Katz, 1964; Katz and Kahn, 1966) distinction Katz made between in-role and extra-role behaviors in his 1964 book. Task behavior is a set of recurring actions (Katz and Kahn, 1966), which can include fulfilling assigned duties, complying with company rules, and working the expected number of hours in a day (Williams and Anderson, 1991, p. 602). On the other hand, OCB includes a number of different types of behaviors (Organ et al., 2006) that are not included in the job definition, but which without, organizations would not function (Katz and Kahn, 1966). There are several distinctions in types of OCB behaviors. For instance, OCB helping (e.g., orienting new employees, or assisting coworkers with workflow) is considered an affiliative promotive behavior that is cooperative and helps to build relationships, while OCB voice (e.g., speaking up with new or innovative ideas) is an example of challenging promotive behavior, which emphasizes ideas and issues (Van Dyne and LePine, 1998).

We propose that the ideal self would have an impact on both task behavior and extra-role behavior because prior research has found that proactive and future-focused employees engage more frequently in both types of role behaviors (Bergeron et al., 2013). Bergeron et al. (2014) found that professionals with proactive personality engage more frequently in both task behavior and OCB. Also, it has been argued that aspects like the alignment of personal values with those of the organizations,' or the perception from the employee of the companys' Social Responsibility programs, motivates them toward OCB directed toward the organization itself (Ahmad et al., 2020; Islam and Irfan, 2020). Additionally, Strobel et al. (2013) found that employees with a future-oriented mindset were more likely to perform OCB. Both studies concluded that more frequent engagement of in-role and extra-role were part of how employees either make their personal vision a reality (Bergeron et al., 2014), or reflected an “employee's desire to make the workplace a better place and to create a positive future for themselves and the organization” (Strobel et al., 2013, p. 830). As such, it would be expected that employees who have included their jobs in their future-oriented ideal self would be more likely to engage more frequently in task behavior and in both types of OCB—helping and voice—as a way to influence and transform their work context toward the vision of their ideal self.

*Hypothesis 3: The ideal self is positively related to task behavior*.
*Hypothesis 4: The ideal self is positively related to OCB helping*.
*Hypothesis 5: The ideal self is positively related to OCB voice*.

## Methods

### Research Design

To answer the research question, this study has been designed to measure the impact of an employee's ideal self within an organizational setting – which implies a complex setting of multiple perspectives from different organizational members. As such, data was collected applying a variation of a 360-degree multi-rater assessment. This included a mix of self-rated and other-rated (i.e., supervisors, colleagues and direct reports) dependent variables. The self-reported measures looked to capture an employee's ideal self and their level of energy and fulfillment both at work and in their life in general. The two measures used, were psychological well-being and job engagement. To look at the impact of an employee's ideal self on their behavior at work, the experiment captured three measures related to the perceived quality of the employee's role fulfillment at work— task behavior (i.e., in-role behavior) and OCB helping and voice (extra role-behavior), which were assessed by collecting responses from a participant-determined set of direct reports, colleagues, and supervisors.

### Sample Participants and Procedure

Data was collected from employees in firms from Costa Rica. To recruit firms in which to collect employee data, a “snowball” sampling technique was applied. While snowball sampling suffers from potential biases of unknown magnitude and direction (Erickson, 1979), controls were built into the firm recruiting process, as well as in the data analysis process. For example, recommendations were received from two different sources and employees from a variety of industries were included in the sample. Five firms provided their support and provided a list of contacts to recruit from their employees.

The sample consisted of 239 mid-level managers—defined as reporting to a manager, having peers of similar hierarchical level, and having direct reports—with a mean age of 41.53 years, and a mean of 14.45 years in the company. 48.1% of the respondents were women (115). 94.5% of the participants had at a minimum a college degree. The 239 managers were recruited from 5 companies with a maximum of 106 from one company and a minimum of 18. The industries of these firms included finance, government, administrative services and production.

The behavioral measurements (i.e., task behavior and OCB) were collected from supervisors, peers and direct reports. After receiving consent and filling out the survey, participants were asked to send a list of at least five individuals, listed as supervisors, peers or direct reports. Of the 239 managers, 183 received ratings (77%, with a mean of 4.21 raters per manager). The supervisor, peer and direct report raters had an average of 13.55 years of experience in the company.

### Data Collection and Analysis

Data collection was done in waves using Qualtrics (Hodge and Kipka, 2012) over a 4 months period. First managers gave consent to participate and filled out their part of the survey. They then received an email requesting a list of supervisors, peers, and direct reports who would be contacted to fill out the behavioral measures of the study. After that, the raters received an email explaining the study and the data collection process. The online self-reported survey had a total of 115 items. The self-reported survey took on average 40 min. The supervisor, peer and direct report survey had 30-items. The other-rated survey took on average 5 min.

### Measures

#### Dependent Variables

*Psychological Well-being*. The PWB measure is the shortened 18 item version (Ryff, 1995) of Ryff's 42-item measure (1989). *Job Engagement*. The job engagement measure is the Rich et al. (2010) scale that proposes to “more fully reflect Kahn's (1990) conceptualization as the degree to which individuals invest their physical, cognitive, and emotional energies into their role performance” (Rich et al., 2010, p. 623). *Task behavior*. For task behavior, we used the 7-item scale from Williams and Anderson (1991) (α = 0.91). *OCB Helping and Voice*. For OCB helping, we used the seven-item unidimensional scale from Podsakoff et al. (1997). For OCB voice, we used the six-item voice sub-scale from Van Dyne and LePine (1998).

#### Independent Variable

*Ideal Self*. The ideal self measure was developed from theory (Boyatzis and Akrivou, 2006). The response options of the 20-item survey are based on a 7-point Likert scale (1 = Strongly disagree to 7 = Strongly agree). The measure has four factors that include holistic, feelings, salience, and mindfulness. Items include “My vision includes my contributions to others and the community,” “I feel inspired by my vision of the future,” “My vision of the future reflects the things most important to me” and “I have a clear vision of my desired future.” The survey starts with an optional written space that allows the participant the opportunity to describe in as much detail their ideal life.

#### Control Variables

We controlled for hope and self-efficacy as these variables are theoretically related to both the ideal self (Boyatzis and Akrivou, 2006) and our dependent variables. *Hope*. The Hope state scale was developed by Snyder et al. (1996). *Self-Efficacy*. The Self-Efficacy scale was developed by Schwarzer and Jerusalem (1993, 1995). Demographic variables to be collected will include participants' age, gender, level of education and tenure in the organization. To control for group differences and some of the biases from the snowball sampling, we controlled for organization. For the other-rated measures, we controlled for number of raters, the ratio of women to men of raters per participant, and the tenure of raters in the organization per participant.

## Results

### Statistical Analysis

We tested our hypotheses using structural equation modeling (SEM) analysis using SPSS AMOS 22 for Windows (Arbuckle, 2014). A SEM analysis allows for a series of dependent relationships to be examined simultaneously and is particularly appropriate when dependent variables become independent variables in a model (Hair et al., 1998). As well, SEM is widely used in the management study, and provides a method to assess the relationships amongst variables comprehensively and provide a transition from exploratory to confirmatory analysis (Hair et al., 1998). Another benefit of SEM is the ability to “represent unobserved concepts in these relationships and account for measurement error in the estimation process” (Hair et al., 1998, p. 584).

Prior to data analysis, the collected data was screened for missing data, outliers, and normality (i.e., skewness and kurtosis). Missing values were <1%. For the other-rated measures of task behavior, OCB helping and OCB voice, after grouping the responses by the employee they were filled out for, the mean was generated for each item. However, to maximize the use of the self-rated data on AMOS, the option for estimating means and intercepts was chosen, as such the analysis was run on the 239 participants using 183 other-rated scores. All items were then screened and no issues of skewness and kurtosis were identified.

Common method bias (CMB) was assessed running an EFA with all the self-rated measures and forcing them into one factor (i.e., Harman's Single-Factor Test) (Podsakoff et al., 2003). If the one factor explained more than 60% of the variance, then CMB is a concern (Podsakoff et al., 2003). After running the EFA, the one factor explained 15% of the data, as such CMB was not found in this data set. Nonetheless, to generate and input the composite scores on AMOS, as recommended by the Harman Test, a Common Latent Factor was generated and linked to each item to remove the variance that could be linked to CMB.

To establish discriminate validity of the constructs, the correlations between factors and square root of the average variance extracted were compared. Discriminant validity was supported as the average variance explained (AVE) for each factor was greater than the correlations between that factors and other factors (Fornell and Larcker, 1981). Convergent validity was assessed calculating the AVE for each factor. All factors have an AVE >0.5 (Fornell and Larcker, 1981).

The SEM model was used to impute all the scales, except for generalized self-efficacy. To generate the final composite for generalized self-efficacy, SPSS was used to calculate the mean composite of the items.

The SEM analysis was first run using the full-saturated model. The model was then modified adding links between error terms using the modification indices generated by AMOS. After adding the links between error terms, non-significant paths were removed one by one, until only significant paths remained.

### Findings

**Figure 1:**
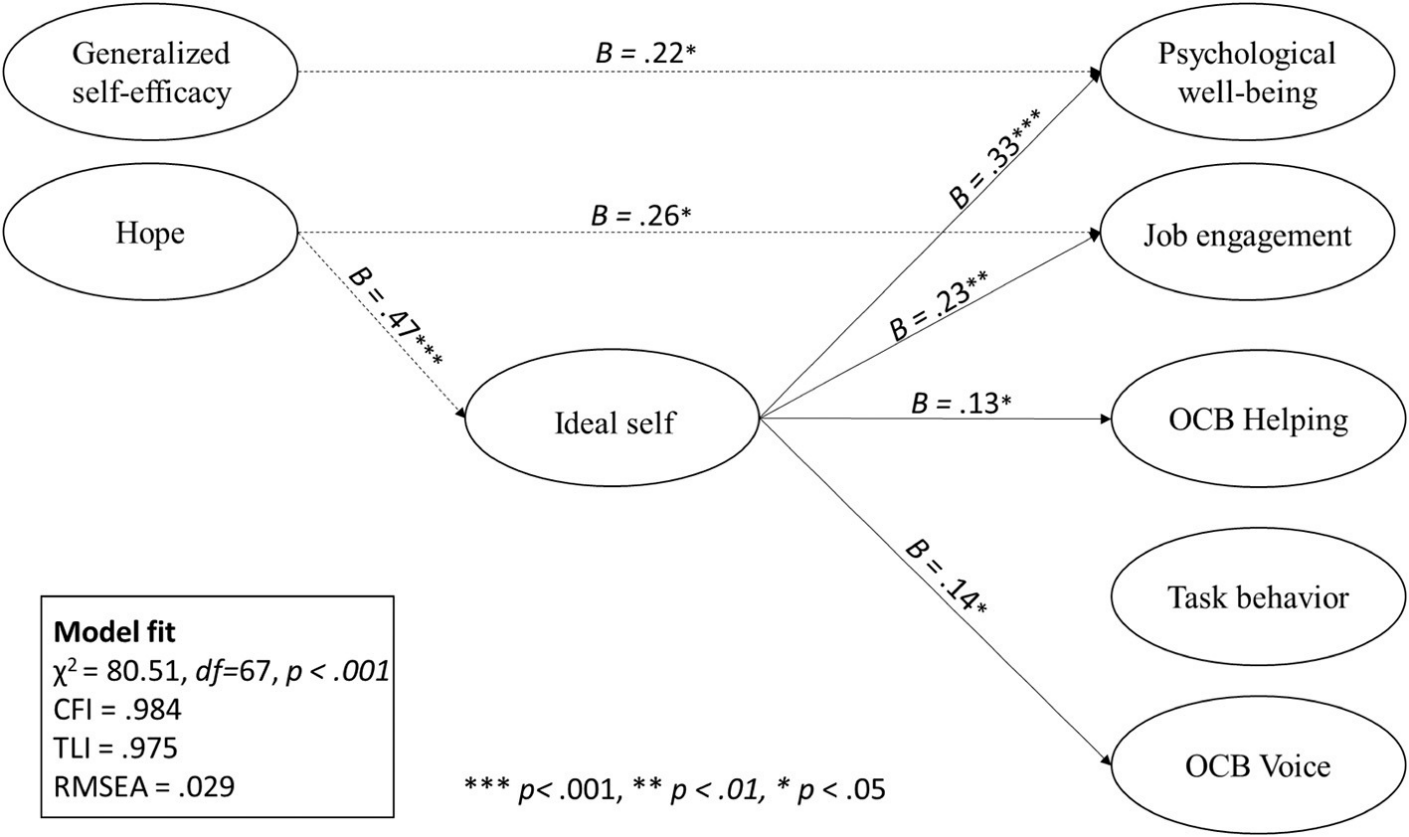
Final model showing significant paths. Demographic control variables not shown for simplicity include age, gender, company, years in company, education, number of raters, ratio of male to female raters, and rater's years in company.

Our model (Figure 1) shows excellent fit with the data (χ^2^ = 80.51; *df* = 67; *p* < 0.001; TLI = 0.975; RMSEA = 0.029; CFI = 0.984). The first two hypotheses focus on the impact of the ideal self on how employees feel at work. Hypothesis 1 proposed that the ideal self has a positive impact on job engagement, while hypothesis 2 proposes that the ideal self has a positive impact on well-being. The results provide evidence for both hypotheses. The ideal self has a positive effect on employee job engagement (β = 0.23; *p* < 0.05) and well-being (β = 0.33; *p* < 0.05).

Hypotheses 3–5 test the impact of the ideal self on employee behavior. Hypothesis 3 proposed that the ideal self has an impact on task behavior. Hypotheses 4 and 5 proposed that the ideal has an impact on the extra-role behaviors of OCB helping and OCB voice, respectively. The results of the analysis did not provide evidence for hypothesis 3. However, the ideal self was found to have a positive effect on both OCB helping (β = 0.13; p < .05) and OCB voice (β = 0.14; *p* < 0.05). These results are summarized in Table 1. Table 2 summarizes the results of the analyzes.

**Table 1:** Summary statistics and correlations.

		**Mean**	**s.d**.	**1**	**2**	**3**	**4**	**5**	**6**	**7**	**8**	**9**	**10**	**11**	**12**	**13**	**14**	**15**	**16**
1	Age	41.53	8.96	1.00															
2	Gender	0.48	0.50	−0.08	1.00														
3	Education	2.38	0.59	0.05	0.05	1.00													
4	Company	3.08	1.05	0.21^**^	−0.12	−0.04	1.00												
5	Years in company	14.45	9.90	0.73^**^	0.04	−0.05	0.20^**^	1.00											
6	Number of raters	4.21	2.24	−0.04	0.05	0.13	−0.16^*^	0.02	1.00										
7	Years in company–raters	13.56	6.52	0.40^**^	0.03	−0.02	0.25^**^	0.49^**^	−0.11	1.00									
8	Raters gender ratio	0.56	0.28	0.01	−0.18^*^	−0.18^*^	0.10	−0.09	−0.19^*^	−0.03	1.00								
9	Generalized self efficacy	3.56	0.37	−0.03	−0.09	−0.05	−0.04	0.07	−0.02	0.08	0.02	1.00							
10	Hope scale	5.76	0.62	−0.02	0.05	0.07	−0.08	0.13^*^	0.00	0.06	−0.07	0.32^**^	1.00						
11	Ideal self	6.38	0.50	0.02	−0.06	−0.06	−0.16^*^	0.09	0.03	0.01	0.00	0.21^**^	0.47^**^	1.00					
12	Psychological well-being	4.73	0.49	−0.05	−0.01	0.02	−0.11	0.03	−0.06	0.08	0.05	0.31^**^	0.34^**^	0.37^**^	1.00				
13	Job engagement	6.40	0.52	0.08	−0.01	−0.09	−0.10	0.13	0.05	−0.03	0.01	0.21^**^	0.40^**^	0.35^**^	0.38^**^	1.00			
14	Task behavior	5.27	0.60	−0.02	0.11	0.16^*^	−0.03	0.02	0.10	0.15^*^	0.10	0.09	0.04	0.07	0.17^*^	−0.03	1.00		
15	OCB helping	4.97	0.64	0.05	0.01	0.06	−0.04	0.08	−0.03	0.05	0.11	0.04	0.06	0.16^*^	0.19^**^	0.03	0.59^**^	1.00	
16	OCB voice	4.99	0.57	0.06	−0.04	0.24^**^	0.04	0.10	0.04	0.07	0.02	0.05	0.07	0.15^*^	0.17^*^	0.08	0.55^**^	0.68^**^	1.00

*Two-tailed significance*:

* *p < 0.05*;

** *p < 0.01; Gender coded as men = 0, women = 1; Cronbach's alpha are shown on the diagonal for scale variables*.

**Table 2:** Summary model of results.

	**Hypothesis**	**Results**	**Support**
H1	The ideal self has a positive relationship with job engagement.	*B =* 0.23^**^	Yes
H2	The ideal self has a positive impact on psychological well-being.	*B* = 0.33^***^	
H3	The ideal self has a positive relationship with task behavior.	Not significant	No
H4	The ideal self has a positive relationship with OCB helping.	*B =* 0.13^*^	Yes
H5	The ideal self has a positive relationship with OCB voice.	*B =* 0.14^*^	Yes

*Standardized betas are reported*.

*** *p < 0.001*,

** *p < 0.01*,

* *p < 0.05*.

## Discussion

This study tested Boyatzis and Akrivou (2006) propositions that employees with a healthy and robust (i.e., meaningful and useful) ideal self that has been articulated and has integrated the components of a person's desired life and future expressed – including their work and career – will be more engaged, feel better about their lives, and will have better performance at work. This ideal self is a vision that embeds an individual's will with positive affect and generates both energy and inspiration that works in self-regulation to “guide actions and decisions in a direction which ensures deeper self-satisfaction […] and […] toward either: the emergence of a new state of being or the maintenance” of characteristics that are consistent with that vision of the ideal self (Boyatzis and Akrivou, 2006, p. 626).

To test these propositions, an SEM model was hypothesized using the recent quantitative measure of the ideal self (Buse and Bilimoria, 2014) that looks to assess the clarity, meaningfulness, and inclusion of the ideal self. This model linked the inspirational and motivational energy and positivity of the ideal self to quantitative measures of employee engagement, psychological well-being, task behavior, OCB helping and OCB voice. In the model, it was hypothesized that employees who had a healthier and more robust ideal self would feel more engaged by their work and would feel better and more positive about their lives. It was also hypothesized that a healthier and more robust ideal self would impact how employees performed in their jobs through their in-role and extra-role behaviors.

The results of the SEM analysis provided support for several of these hypotheses. First and foremost, evidence was found to support the proposition that a more inspiring and representative ideal self had a positive and significant impact on both job engagement and well-being. Evidence was also found for the impact of the ideal self on how employees were evaluated by direct reports, coworkers and supervisors on OCB helping and voice. Finally, support was not found for the impact of the ideal self in task behavior.

### Theoretical Contributions

This study makes several contributions to the literature on employee motivation, self-regulation and the ideal self: (1) it provides evidence for the impact of the ideal self in the engagement of employees in their work; (2) it provides evidence for the direct impact of the ideal self on how employees feel about the quality of their lives; (3) this study provides evidence for the potential employee performance benefits that can be realized when employees have a more inclusive and inspiring ideal self as a self-regulating guide; and (4) this study also contributes to the research on the meaningfulness of work by adding the ideal self as one of the mechanisms that helps employees identify, construct and discover purpose and meaning in their work.

#### The Ideal Self and Employee Engagement

One contribution this study makes is finding evidence for the link between the ideal self and employee engagement. While there are many theoretical propositions (Boyatzis and Akrivou, 2006) to link the ideal self to employee engagement, this study is the first to directly test the relationship between employee ideal self—or indeed any self-representation—to engagement. Results found that the inclusiveness and inspiration of an employee's ideal self is an antecedent to employee engagement. As evident in the Gallup employee surveys mentioned earlier in the introduction, modern day organizations have an employee engagement problem, and they do not know what to do about it. But even though shifts in the work relationship have changed how employees and employers relate to each other, engagement is not a new problem. In his Theory X and Theory Y framing, McGregor (1960) surfaced assumptions held by both managers and employees that limited – or enhanced – the possibility of employee engagement. He proposed that organizations were not designed to foster employee engagement, indeed management can only create conditions for an employee to find engagement and meaning, or discourage it by failing to create those conditions (McGregor, 1960). While current studies on employee engagement have identified dozens of individual and organizational level antecedents to employee engagement (Simpson, 2009; Bakker et al., 2011; Christian et al., 2011; Wollard and Shuck, 2011; Islam and Tariq, 2018; Islam et al., 2019), these antecedents “are not process dependent, but rather functions that usher in the conditions for the state of engagement to develop” (Wollard and Shuck, 2011, p. 436), in many ways validating McGregor's proposition. As such, identifying an antecedent that may be a part of a process to help employees to feel engagement at work holds considerable value for both researchers and practitioners (Wollard and Shuck, 2011). As part of the intentional change coaching process (Boyatzis, 2006), the ideal self holds such promise.

The meaningfulness and utility of an employee's ideal self varies from individual to individual, and is subject to considerable self and organizational awareness (Boyatzis and Akrivou, 2006). For instance, the ideal self tested in this study was self-constructed – captured through a survey questionnaire – and based solely on the perceptions and assumptions of employees about their work and their relationship to and future in the organization. As such, employees who were more creative, or had better relationships with their bosses and had more input in their role at work were more likely to find ways through which their jobs and their ideal future converge.

Future research directions could explore the impact of an ideal self intervention – which helps in articulation, salience and integration of the ideal self – as part of the intentional change coaching process, on employee engagement. It is possible that researchers could find that a coaching for intentional change intervention can provide considerable help for employees and organizations in aligning work roles toward realizing both organizational objectives and the employee's ideal self, and helping more employees find engagement in their work.

A second contribution from this study relates to the research on employee ideal self and well-being. Most of the research linking work and well-being has approached the question of well-being at work from a decidedly deficit-focused framing. For instance, considerable attention has been given to the aspects of work that lead to stress and reduce employee well-being (Griffin and Clarke, 2011; Monnot and Beehr, 2014). As such, there has been relatively less research that has directly looked at the role work can play in enhancing well-being and meaningfulness in the life of employees (Monnot and Beehr, 2014).

The current study proposed that employees with a healthier and more robust ideal self have visualized ways in which their job relates or helps them to realize their ideal self. Other studies support why the ideal self would lead to employee well-being. For instance, Carver and Scheier (1999) found a positive link between approach goals and well-being. Brunstein et al. (1998) found that motive and goal alignment was linked to well-being. Several researchers have found a link between autonomy in goals and well-being (Sheldon and Kasser, 1998; Sheldon and Elliot, 1999; Ryan and Deci, 2000), as well as meaningfulness of goals and well-being (McGregor and Little, 1998). Indeed in many ways, the realization of one's ideal self is the defining characteristic of well-being (Ryan and Deci, 2001). As such, while the ideal self plays a substantial role in well-being, no study has directly tested this relationship in the work context. Our results provide clear evidence that when an employee has included their job in their ideal self, employees feel more satisfied and more engaged by the existential challenges of life (i.e., PWB). Additional to previous research relating psychological well-being to different factors and antecedents such as emotional intelligence (Kamboj and Garg, 2021) and organizational error tolerance (Wang et al., 2020), the ideal self comes to create a more comprehensive picture of what leads to employee well-being.

These results also contribute to research on coaching employees for personal and professional development. Boyatzis et al. (2012) proposed that using the intentional change coaching process (or coaching with compassion) for employee development would be positively related to employee psychological well-being. Results from this study seem to at least provide partial validation for this proposition. The first step in the intentional change coaching process is to help individuals articulate a more meaningful and holistic ideal self. The intentional change coaching process leads individuals to become more aware of their strengths and opportunities, helping them to self-regulate their behavior at work to realize or maintain their ideal self. Future research studies may help to further validate Boyatzis et al. (2012) proposition.

The results of the SEM analysis provided evidence for the proposition that an articulated, healthy and robust ideal self works as “an executive motivational force that monitors and guides behavior in a direction that ensures the emergence of a new state of being of self-actualization” (Boyatzis and Akrivou, 2006, p. 625). This proposition was tested using employee performance in both in-role (task behavior) and extra-role (OCB helping and OCB voice). These results join other studies that have found the self-regulating impact of other future-oriented constructs in employee behaviors. Strauss et al. (2012) found that the salience of an employee's *future work self* – a bounded work-focused future possible self – had a motivating impact on proactive career behavior, which included proactive skill development, networking, career consultation and career planning (Strauss et al., 2012, p. 585). These findings lead Strauss et al. to conclude that accessible representations of the ideal self provide meaning for *future focused* behaviors at work (2012). Strobel et al. (2013) also found that employees with a *future-oriented* mindset were more likely to perform OCB. Strobel et al. proposed that positive extra-role behaviors reflected an “employee's desire to make the workplace a better place and to create a positive future for themselves and the organization” (Strobel et al., 2013, p. 830).

The results from this study extend those earlier findings in two significant ways. First, this study tested the impact of the *ideal self*, which is considered one of the core self-representations in self-regulation (Higgins, 1987; Carver and Scheier, 1991; Boyatzis and Akrivou, 2006). While the ideal self seems to differ depending to some personalities or individual dispositions (e.g., approach vs. avoidance predilection: Higgins et al., 1994), its intentional development—for instance as part of a coaching process—allows for the construction of a vision that can create consistency across different aspects and components of one's ideal life. This broader and inclusive vision is particularly important for identifying inconsistencies that may arise between tangential future selves. Furthermore, once constructed, a broader and inclusive vision of the ideal self can help an individual discern between the *ought self* and the ideal self. The *ought self* – developed from ideas that an individual has about what others want them to be – can be mistaken for the ideal self, and can come into conflict with the ideal self (Higgins, 1987; Higgins et al., 1994; Brockner and Higgins, 2001; Boyatzis et al., 2012). By using the ideal self in this study, the likelihood of that confusion is reduced. Indeed, encouraging employees to think in a more holistic manner about their future self may be particularly important at work. Role theory (Katz and Kahn, 1966) explains that job roles (such as *future work self*) are socially constructed from the expressed and assumed demands and needs of supervisors, coworkers and direct reports (Katz and Kahn, 1966). This context may potentially lead to greater clouding and conflict between the *ought* and the ideal self.

Second by incorporating measures of an employee's job performance in an integrated work context (i.e., OCB help and OCB voice), these results display the direct benefits available to organizations when an employee is allowed – or encouraged – to visualize their holistic ideal self at work. Encouraging an employee to develop their ideal self in a work context may hold considerable benefits for organizations beyond the findings of this study. For instance, Cable et al. (2013) found that new employees who were encouraged to express their authentic self (a self-representation of their *best self*) – as opposed to being socialized to develop pride solely from being a part of their new organization – led to both greater customer satisfaction and employee retention. Future studies could test the organizational benefits of encouraging new incoming hires to dream about their future as they join an organization.

Finally, this study contributes to the mechanisms of how employees find meaningfulness in their work. Rosso et al. (2010) noted that the self is a source of meaning at work, but research has primarily focused on singular components of the self, such as values, motivations and beliefs about work. However, there has been very limited research on the ideal self in the context of work. Nonetheless, the findings of this study display that this relationship holds much promise for the employee and organizations. Research on meaningfulness at work has primarily explained the state of meaningfulness generated from congruence between the disposition of the employee and environmental circumstances (Barrick et al., 2013). This study is one of the first studies to incorporate the ideal self to assess its impact of feelings and behaviors related to meaningfulness at work. The model and the results of the study point to the impact of an employee's ideal self as a path toward identifying congruence between disposition of the employee and environmental circumstances.

As such, these findings point toward the benefits that both employees and organizations can realize from promoting their employees to develop a vision of their ideal future. These findings also contribute to McGregor (1960) notion of integration in several ways. Crafting a vision of the ideal self – as part of a coaching process – would help employees to have more self-awareness of both their base and higher-level needs, and provide a structure around which an employee can better verbalize these needs and goals to their managers. Knowing one's true needs and goals are primary to any integrative negotiation strategy. An employee that has not spent time identifying their higher-level needs would have little input in the design and construction of an integrated job negotiation.

### Practical Implications

The most direct practical implications from this study relate to coaching processes within organizations. While coaching processes generally include a conversation about the future, generally the coaching processes initiates with a 360-degree feedback process (Passarelli, 2015). These types of conversations have been found to trigger the Sympathetic Nervous System and feelings of shame, guilt and anxiety in individuals (Jack et al., 2013). The findings from this study provide further evidence for the need to include an articulation of the ideal self using the questionnaire. This could be incorporated in human resource management practices to develop work engagement (Bakker, 2017), as well as part of a job crafting intervention to help employees use their individual strength in their jobs (Tims et al., 2015; Meyers et al., 2019). The benefits of meaningful work becomes available to organizations when they apply a coaching process that focuses on an employee's strengths and the development of their personal vision (Boyatzis et al., 2012; Grant et al., 2013; Passarelli, 2015; Theeboom et al., 2015).

In a more substantial sense, one practical contribution from this study relates to whether organizations should invest in the coaching process for employees, particularly when what may emerge from the process may lead an employee to leave the firm. In other words, why would a firm want to invest in coaching employees around their ideal self, when there is a possibility that the process of expressing the ideal self will actually drive the employee to find another career path that is more aligned with their ideal self. This study helps to address this scenario in several ways. First, the realization that an employee wants to change their career is something that happens naturally in organizations, with or without coaching. It has been reported that 59% of the US workforce is searching for a new job (Nguyen et al., 2019). Perhaps the opportunity to speak about these issues with a coach can help ease and structure the transition for the employee and the organization. But perhaps, and probably more important, coaching around the ideal self can help the employee find ways in which their current job helps them develop toward that ideal self, and facilitate–albeit limited–employee engagement for their current job. The focus may best be framed as not for how long an employee's tenure in the organization will be, but how impactful that employee's engagement with their role will be for as long as they will be with the organization.

### Limitations

There are several limitations in this study. First the study suffers from a relatively small sample size, limiting generalizability. However, the data comes from five different sites, and none is from a student sample. Furthermore, the ideal self, employee engagement, and psychological well-being are self-reported. While the relationships in the model follow the proposed theoretical direction, cross sectional data limits the confidence of the causal direction of the relationships. Cross sectional data is also subject to common method bias. Common method bias was controlled for statistically in the SEM analysis. Finally, all the behavioral measures were captured from other-raters (i.e., supervisors, coworkers, and direct reports). The other-raters were recommended by the participants themselves. This data is subject to the biases from which all 360-degree evaluations suffer.

### Direction for Future Research

Several future research directions have been mentioned earlier. First, the results from this study validate the ideal self and its role in how employees feel and behave at work. As such, an experimental design study testing the impact of an ideal self intervention on employee performance, engagement, and well-being would help to further understand the mechanisms of the ideal self at work. Also testing the impact boundaries of the ideal self may also provide input in its mechanism. For instance, would an ideal self intervention have a significant impact on an employee who has already included their work role within their ideal self? While it may not have a significant performance impact, this intervention may have a significant impact in relationship measures, OCB, and a negative impact in both job burnout and employee turnover.

Another research direction may look at long-term impact of employee ideal self both within the organization and over the career of an individual. Such a study might help to understand the developmental phases of the ideal self, and the role of multiple “ideal selves” in career success and life satisfaction. A third research direction could focus on the role of the ideal self as part of a coaching for intentional change intervention in employee development.

## Conclusion

In the more transactional work relationship, perhaps to talk about the meaningfulness of work is old fashioned or even extravagant. Indeed, when it is found in the work place it seems to be an unintended positive externality. But just as how bees in honey farms benefit nearby almond trees with pollination, employees who find meaning in their work benefit organizations and coworkers. Ironically, the more transactional work relationship may finally provide the opportunity for better integration between an individual's higher-order needs (McGregor, 1960) and the organization. As individuals become less dependent on one organization in the long-term, to compete for talent organizations have adapted. Sustainability is now a recruiting tool for organizations. Helping employees to dream and better understand themselves through their ideal self may be the key to engagement and meaningfulness, and in the future may be a key tool for recruiting talent.

## Data Availability Statement

The raw data supporting the conclusions of this article will be made available by the authors, without undue reservation.

## Ethics Statement

The studies involving human participants were reviewed and approved by Case Western Reserve University IRB. The patients/participants provided their written informed consent to participate in this study.

## Author Contributions

All authors have contributed to this paper. The paper was grounded on Hector Martinez's dissertation, which was supervised by RB, who contributed in theory, design, and editing. KR contributed to the writing and editing of the original dissertation into this version. SR-C provided help in editing and formatting this paper for submission to Frontiers in Psychology.

## Conflict of Interest

The authors declare that the research was conducted in the absence of any commercial or financial relationships that could be construed as a potential conflict of interest.
